# Depression, Anxiety and Somatic Distress in Domestic and International Undergraduate Medical Students in Kazakhstan

**Published:** 2018-06

**Authors:** Zhanar IBRAYEVA, Daulet ALDYNGUROV, Ayan MYSSAYEV, Serik MEIRMANOV, Marat ZHANASPAYEV, Zaituna KHISMETOVA, Zhanna MUZDUBAYEVA, Erbol SMAIL, Oksana YURKOVSKAYA, Lyudmila PIVINA, Yuliya SEMENOVA

**Affiliations:** 1. Dept. of Public Health, Semey State Medical University, Semey, Kazakhstan; 2. Medical Center Hospital of President’s Affair Administration of the Republic of Kazakhstan, Astana, Kazakhstan; 3. Ritsumeikan Asia Pacific University, Beppu, Japan

## Dear Editor-in-Chief

Globally depression presents one of the major public health problems and the WHO predicts it to be the second leading cause of disability worldwide by 2020 (1). The period of transition to university could be stressful for future doctors as students are learning to cope with increased academic pressures (2). Usually, at this time, they are exposed to many newfound stressors that can promote the onset of mental health problems and their somatization (3).

The aim of this study was to screen for the rates of anxiety, depression and somatic distress among first-year medical students in three out of five medical schools of Kazakhstan.

During 2015–2016 academic year we surveyed 1478 first-year medical students, aged 16–30 yr, from the following medical schools: Semey State Medical University, Karaganda State Medical University and Kazakh National Medical University in Almaty. Seven hundred and fifty students from India and 728 students from Kazakhstan completed the self-administered questionnaires anonymously. They were asked to answer demographic questions and Patient Health Questionnaire 9 (PHQ-9), 7-item Generalized Anxiety Disorder (GAD-7), and Patient Health Questionnaire 15 (PHQ 15). Response options were based on a four-point Likert scale. For each disorder total scores of 10 or higher were generally considered clinically significant, signaling a need for additional evaluation and possible intervention. The Ethics Committee of Semey State Medical University approved the study. Student participation was voluntary and information was collected anonymously. Confidentiality of personal data was ensured throughout the study.

The prevalence rate of depression, for a cut-off of 10, was 33.2% (491/1478). Suicidal ideation, represented by Question 9 on the PHQ-9 scale, was attributed to 10.5% (155/1478) of students. Of these, 41.9% (65/155) were domestic students and 58.1% (90/155) were international students. Interestingly, of domestic students who expressed suicidal thoughts the majority – 78.5% (51/65) – were females, while in a bunch of international students these were predominantly males – 80% (72/90).

The prevalence rate of anxiety, for a cut-off of 10, was 22% (325/1478). Female students demonstrated higher rates of anxiety: 30% (190/634) as compared to 16% (135/844) of males.

As for somatic distress, 36.1% (533/1478) of the undergraduate medical students were identified to have PHQ-15 score ≥10. Generally, somatic distress was observed in 24.8% (209/844) of male students and in 51.1% (324/634) of female students. Distribution of PHQ-15, GAD-7 and PHQ-9 scores in a study population is presented in Table 1.

**Table 1:** Distribution of PHQ-15, GAD-7 and PHQ-9 scores in medical students

***Severity score***	***PHQ-15***		***GAD-7***		***PHQ-9***	
	**International students N (%)**	**Domestic students N (%)**	**International students N (%)**	**Domestic students N (%)**	**International students N (%)**	**Domestic students N (%)**
0–4 none	166 (22.1)	50 (6.9)	307 (40.9)	199 (27.3)	271 (36.1)	82 (11.3)
5–9 mild	372 (49.6)	357 (49.0)	285 (38.0)	362 (49.7)	260 (34.7)	374 (51.4)
10–14 moderate	146 (19.5)	240 (33.0)	114 (15.2)	120 (16.5)	151 (20.1)	188 (25.8)
15–19 moderately severe	55 (7.3)	66 (9.1)	31 (4.1)	34 (4.7)	60 (8.0)	59 (8.1)
≥20 severe	11 (1.5)	15 (2.0)	13 (1.8)	13 (1.8)	8 (1.1)	25 (3.4)

Basically, undergraduate medical students represent a particularly vulnerable population because they need to cope with many newfound stressors. For this reason, they are prone to developing mental distress but have little ability to seek treatment for it. The depression rate in our study sample was 33.2%, which corresponds with published data (4). The prevalence of suicidal ideation (10.5%) was lower than what is reported in the literature (around 20%) (4). Still, it could be considered high since a substantial proportion of people expressing suicidal thoughts accounts for half of lethal or nonlethal suicide attempts (5). This is why mental health services need to become available to undergraduate students so that future medical professionals could lead productive and successful lives.

A significant increase in depression, anxiety and somatic distress rates in females is consistent with published literature (6). However, our hypothesis that international students have higher prevalence of depression was not supported by our data. Although some medical schools in Kazakhstan had already implemented certain measures to address emotional well-being of their undergraduate trainees (7), our study shows that medical students still have high rates of positive screens for mental distress.

Our study had certain advantages. The nature of our survey enabled to interview a very large population (1478 students in total) and to cover all medical schools providing training to students from India. However, our study had several disadvantages. The non-prospective nature did not allow us to establish causal determination for possible predictors. In addition, while PHQ-9, GAD-7, and PHQ-15 are well-validated tools that measure the degree to which a patient suffers from the symptoms of mental distress, scores of 10 or more are not synonymous with a diagnosis of depression, anxiety, and somatic distress. Moreover, we could not differentiate psychologically related somatic symptoms from physical symptoms caused by the presence of somatic disorder.

## References

[1] Murray CJ Lopez AD (1996). Evidence-based health policy--lessons from the Global Burden of Disease Study. Science, 274(5288):740–3. 8966556 10.1126/science.274.5288.740

[2] Rutter M Sroufe LA (2000). Developmental psychopathology: concepts and challenges. Dev Psychopathol, 12(3):265–96. 11014739 10.1017/s0954579400003023

[3] Dyson R Renk K (2006). Freshmen adaptation to university life: depressive symptoms, stress, and coping. J Clin Psychol, 62(10):1231–44. 16810671 10.1002/jclp.20295

[4] Naja WJ Kansoun AH Haddad RS (2016). Prevalence of depression in medical students at the Lebanese university and exploring its correlation with Facebook relevance: a questionnaire study. JMIR Res Protoc, 5(2): e96. 27246394 10.2196/resprot.4551 PMC4908302

[5] Simon GE Rutter CM Peterson D (2013). Does response on the PHQ-9 Depression Questionnaire predict subsequent suicide attempt or suicide death? Psychiatr Serv, 64(12):1195–202. 24036589 10.1176/appi.ps.201200587 PMC4086215

[6] Yelissinova N Grjibovski AM Yelissinova A (2015). Sociodemographic factors associated with infant abandonment in maternity hospitals in Kazakhstan: A case-control study. Public Health, 129(7): 1010–1013. 25986952 10.1016/j.puhe.2015.04.009

[7] Ibraieva ZhB Semenova YuM Aldingurov DK (2016). The satisfaction of foreign students with social cultural support in the medical university of the Republic of Kazakhstan. Probl Sotsialnoi Gig Zdravookhranenniiai Istor Med, 24(1):39–44. [Russian]. 10.1016/0869-866X-2016-1-39-43 31491066

